# A machine-learning informed circulating microbial DNA signature for early diagnosis of esophageal adenocarcinoma

**DOI:** 10.1080/19490976.2025.2604334

**Published:** 2025-12-24

**Authors:** Yuan Li, Caiming Xu, Hyun Park, Ashten N. Omstead, Muhammad Anees, Chris Sherry, Alisha F. Khan, Erin Grayhack, Benny Weksler, Patrick Wagner, David L. Bartlett, Stephen J. Meltzer, Ali H. Zaidi, Ajay Goel

**Affiliations:** a Department of Molecular Diagnostics and Experimental Therapeutics, Beckman Research Institute of City of Hope, Biomedical Research Center, Monrovia, California, USA; b Department of Clinical Laboratory, Yangpu Hospital, Tongji University School of Medicine, Shanghai, People's Republic of China; c Department of Clinical Laboratory, Shanghai Children's Hospital, Shanghai Jiaotong University, Shanghai, People's Republic of China; d Department of General Surgery, The First Affiliated Hospital of Dalian Medical University, Dalian, People's Republic of China; e Allegheny Health Network Cancer Institute, Allegheny Health Network, Pittsburgh, PA, USA; f Division of Thoracic Surgery, Department of Cardiothoracic Surgery, Allegheny Health Network, Pittsburgh, PA, USA; g Division of Gastroenterology and Hepatology, Department of Medicine and Oncology, Sidney Kimmel Comprehensive Cancer Center, Johns Hopkins University School of Medicine, Baltimore, MD, USA; h City of Hope Comprehensive Cancer Center, Duarte, California, USA

**Keywords:** Esophageal adenocarcinoma, Barrett's esophagus, aarly diagnostic biomarker, circulating microbiome DNA, XGBoost algorithm

## Abstract

Esophageal adenocarcinoma (EAC) has seen a dramatic rise in incidence in developed countries over the past three decades. Early detection of its precursors—gastroesophageal reflux disease (GERD), Barrett's esophagus (BE), and high-grade dysplasia (HGD) is critical for cancer prevention. This study presents the development and validation of a novel liquid biopsy assay based on circulating microbial DNA (cmDNA) for the early detection of EAC and HGD. Using metagenomic sequencing, we identified significant differences in microbial diversity and composition between EAC and HGD patients, as well as between BE and GERD patients. A total of 46 microbial candidates in tissue and 419 in serum were upregulated in EAC & HGD, with 11 consistently elevated in both sample types. Following qRT-PCR validation and LASSO regression, a 6-marker cmDNA panel was selected. This signature was incorporated into a diagnostic model trained with the XGBoost algorithm, achieving an AUC of 0.93 in the training cohort (52 HGD & EAC cases vs. 54 BE & GERD controls). Importantly, the model demonstrated robust performance in an independent testing cohort (23 HGD & EAC cases vs. 22 BE & GERD controls), yielding AUCs of 0.91 for EAC and 0.88 for HGD. These findings highlight the diagnostic potential of cmDNA-based profiling and support its utility as a minimally invasive, accurate, and generalizable tool for early detection of esophageal adenocarcinoma.

## Introduction

Esophageal cancer (EC) poses a significant global health challenge, ranking as the seventh most common cancer and the sixth leading cause of cancer-associated mortality worldwide. By 2040, it is projected that EC will account for approximately 957,000 new cases and 880,000 deaths.^1^ Esophageal adenocarcinoma (EAC) is indeed the most prevalent type of EC in Western countries, and its incidence is significantly increasing, primarily attributed to gastroesophageal reflux disease (GERD) and the development of Barrett's esophagus (BE).^2^ BE is a well-known preneoplastic lesion for EAC, characterized by an archetypal metaplastic change in the distal esophagus.^3^ The relative risk of developing EAC in patients with BE is 11.3 (CI_95%_, 8.8–14.4) compared to the general population.^4^ BE can progress to low-grade dysplasia (LGD), high-grade dysplasia (HGD), and ultimately EAC, typically over a span of about 20 years.^5^ This prolonged period theoretically provides a window of opportunity for implementing cancer prevention strategies. Unfortunately, because the EAC lesions are located in the submucosa near lymphatic drainage, this malignancy is prone to metastasis. Symptoms of obstruction, such as difficulty swallowing and weight loss, typically appear late and only after metastasis. As a result, between 60% and 80% of cases are diagnosed at an advanced stage.^6^ Compared with early diagnosis, the 5-year survival rate for patients with late disease detection plummets from 40%–60% to 5%–16%,^7^ with a median survival time of less than 1 year. This highlights the critical importance of early diagnosis to improve survival and better manage patients affected by this malignancy.

A prospective study demonstrated that endoscopic surveillance of patients with BE led to earlier detection of EAC vis-à-vis the general population.^8^ Another nationwide study from Denmark reported an adenocarcinoma incidence of 5.1 per 1,000 person-years in patients with LGD at the initial endoscopy, compared to 1.0 per 1,000 person-years in those without dysplasia.^4^ Moreover, the American Gastroenterological Association recommends immediate endoscopic eradication therapy rather than surveillance for patients with HGD in BE.^9^ These findings underscore the critical importance of timely detection and intervention for individuals with high-grade dysplasia (HGD) and early-stage esophageal adenocarcinoma (EAC). However, the effectiveness of endoscopic surveillance is limited by low patient compliance and high healthcare costs. In this context, incorporating noninvasive diagnostic approaches could significantly enhance the accessibility and acceptance of current screening strategies. One such method is the Cytosponge, which collects esophageal epithelial cells via a swallowed sponge to detect trefoil factor 3. It has shown promising performance, with sensitivity ranging from 73.3% to 90.0% depending on the BE segment length, and a specificity of 93.8% for BE detection.^10^ In addition, recent studies of plasma cfDNA methylation markers in esophageal cancer have demonstrated their ability to distinguish cancers from healthy individuals and benign esophageal diseases, achieving sensitivities of approximately 75% and specificities exceeding 94%. However, their utility in early-stage disease (stage 0–II) appears limited, with a reported sensitivity of only 58.8%.^11^ Although blood-based testing methods are currently limited, they hold promise as complementary screening tools, offering a minimally invasive and easily repeatable alternative that could enhance participation in surveillance efforts.

Cancer-related circulating microbial DNA (cmDNA) has catalyzed novel research into cancer–microbiome interactions. It reflects the overall microbial burden, as well as the complex interplay between the microbiome, tumors, and the immune system in cancer patients.^12^ Notably, multiple studies have demonstrated consistent alterations in the esophageal microbiota during the disease progression from GERD to EAC. Prior evidence indicates a predominance of gram-negative bacteria in the esophageal microbiome of patients with GERD or BE, which may contribute to chronic inflammation and a pro-tumorigenic microenvironment.^13^,^14^ A longitudinal study involving 100 individuals from diverse ethnic backgrounds in Australia revealed distinct, consistent shifts in esophageal microbial composition as the disease progressed from GERD to metaplasia, driven in part by the enrichment of taxa such as *Campylobacter.*^15^ EAC, the clinical potential of cmDNA has been highlighted in lung cancer, where whole-genome sequencing of plasma from patients and healthy controls identified distinct microbial profiles. A diagnostic model derived from these data achieved an AUC of 93.2% with high sensitivity, including the accurate detection of early-stage tumors and precise prediction of recurrence.^16^ Moreover, a recent analysis of surgical resections from EAC patients revealed that the microbial richness and diversity in tumor-associated microbiota differ markedly from those observed in non-dysplastic BE and normal esophageal tissue.^17^ Collectively, these findings suggest that cmDNA may provide valuable insights for the development of noninvasive diagnostic tools and early detection strategies for EAC.

In this study, we used metagenomic sequencing to characterize microbiome profiles in neoplastic tissues and serum samples from patients with gastroesophageal reflux disease (GERD), Barrett's esophagus (BE), high-grade dysplasia (HGD), and esophageal adenocarcinoma (EAC). Distinct microbial species were enriched among the high-risk patients and low-risk individuals. Using extensive data-informatics approaches, including Linear Discriminant Analysis Effect Size (LEfSe), we identified a panel of cmDNA biomarkers that robustly discriminate between high-risk patients and benign controls. Finally, we successfully established a cmDNA-based liquid biopsy assay that can accurately identify patients with HGD/EAC. This non-invasive assay represents a novel approach that complements existing endoscopic surveillance strategies for the early detection of EAC.

## Materials and methods

### Study design and sample collection

All participants provided informed written consent, and the study protocol was approved by the Institutional Review Board of the Allegheny Health Network (AHN, Pittsburgh, PA, USA) and Johns Hopkins University (Baltimore, MD, USA). This study consisted of three phases: a systematic and comprehensive discovery phase, during which the cmDNA biomarkers were identified from metagenomic sequencing data; a training phase, during which the qRT-PCR-based assay was established; and a testing phase, during which the assay was validated (Figure S1). To identify tissue microbiome-derived specific cmDNA, we performed metagenomic sequencing of tissue specimens from a cohort of 81 patients, including 51 with EAC, 10 with HGD, 10 with BE, and 10 with GERD (Supplementary Table 1). Furthermore, we performed metagenomic analysis of paired serum samples from the same patients, along with additional samples from 13 cases of GERD, 27 cases of BE, 8 cases of HGD, and 5 cases of EAC (Supplementary Table 2). All participants were recruited at AHN between 2016 and 2018 for an observational study. The clinical characteristics of the study participants are outlined in Supplementary Table 1 for tissue data and Supplementary Table 2 for serum data. Following the discovery phase, qRT-PCR was performed to assess the expression levels of candidates for distinguishing HGD (*n* = 13) and EAC (*n* = 62) from BE (*n* = 76) and GERD (*n* = 0). In the Johns Hopkins cohort, samples were collected between 2002 and 2004. The AHN cohort consisted of subjects enrolled at the Esophageal and Lung Institute between 2013 and 2018 as part of an observational study. The AHN cohort comprised 13 patients with HGD, 24 with EAC, and 39 with BE, whereas the Johns Hopkins cohort comprised 38 patients with EAC and 37 with BE. All patients were randomly assigned to either the training or validation cohort. Detailed clinical characteristics of individuals in both cohorts are provided in Supplementary Table 3.

### DNA extraction

The tissues were microdissected from 10-μm-thick formalin-fixed paraffin-embedded (FFPE) sections derived from surgically resected tumors or biopsy specimens. Subsequently, genomic DNA was extracted from the microdissected tissues using an AllPrep DNA/RNA FFPE Kit (Qiagen, Hilden, Germany) according to the manufacturer's instructions.

Serum was separated from peripheral blood samples collected from each subject, aliquoted into sterile microcentrifuge tubes, and stored at −80 °C until further use. DNA from serum was extracted using the PowerSoil Pro Kit (Qiagen) on the QIAcube HT automated platform, which incorporated PowerBead Pro Plates to facilitate high-throughput processing. The library was qualitatively assessed using the Quant-iT PicoGreen dsDNA Assay Kit (Invitrogen, Waltham, MA, USA) and the High Sensitivity DNA kit (Agilent, Santa Clara, CA, USA) on an Agilent 2100 Bioanalyzer.

### DNA library construction

Using a modified version of Illumina's Nextera Library Prep Kit (San Diego, CA, USA), we prepared DNA sequencing libraries. For shallow metagenomic analysis (BoosterShot, ~2 million reads per sample), paired-end sequencing (2 × 150 bp) was conducted on a NextSeq instrument with the 500/550 High Output v2 Kit (Illumina). All sequencing was performed by Diversigen (Houston, TX, USA).

### Sequencing data filtering and annotation

To ensure data quality, raw sequencing reads were initially processed using Trimmomatic to remove adapter sequences, low-quality bases, duplicates, and reads shorter than 50 bp. To eliminate sequences with low complexity, Kcomplexity was applied using its default parameter settings.^18^ Reads of potential human origin were identified by aligning to the human reference genome(hg38) via the Burrows-Wheeler Aligner (BWA) software and subsequently excluded from downstream analysis.^19^

After alignment, a combination of indicators was used to comprehensively identify potential microbial taxa. Microbiota composition profiles were generated based on quality-controlled forward reads using Kraken v2.1.2 and Bracken v2.6.2, with reference to the k2_pluspf_20210517 database. The species-level count data and relative abundance profiles were subsequently analyzed in R software (version 4.1.0) for statistical interpretation.^20^

### Alpha and beta microbial DNA diversity analysis

Alpha diversity of the microbiota for each subject was evaluated at different data levels within each group using the Vegan package in R (version 2.5.7). To explore microbial community structure across groups, principal component analysis (PCA) was used to visualize sample clustering patterns based on genus- or species-level compositional profiles. The microbiome composition across different groups was statistically evaluated using permutational multivariate analysis of variance (PERMANOVA) and principal component analysis (PCA), both implemented in the Vegan package in R (version 2.5.7). Principal coordinates analysis (PCoA) was conducted using the dudi.pco function in the ade4 package in R (version 1.7.18). Associations between specific microbial taxa (at the genus or species level) and clinical parameters were determined using the LEfSe algorithm.

### Real-time quantitative reverse transcription polymerase chain reaction

Cell-free DNA was isolated from serum samples using the PowerSoil Pro Kit (Qiagen) and high-throughput extraction on the QIAcube HT with Powerbead Pro Plates. Quantification of cmDNA was conducted using the SensiFAST™ SYBR Lo-ROX Kit (Bioline, London, UK) on a QuantStudio 6 Flex Real-Time PCR System (Applied Biosystems, Foster City, CA, USA). Primer sequences corresponding to specific cmDNA targets are provided in Supplementary Table 4. Expression levels were normalized to 16S rRNA, which was used as an internal control.

### Statistical analysis

SPSS Statistics 26 was used to analyze clinical variables statistically and compare them between groups. The eXtreme Gradient Boosting (XGBoost) machine-learning algorithm, a decision-tree-based ensemble technique, was employed to build a classifier using cmDNA candidates. This classifier was subsequently applied to the validation cohort utilizing the ‘xgboost’ package (Version 2.0.3). The XGBoost model was trained over 5000 rounds of boosting using gbtrees, with a maximum tree depth of 3. Additionally, a 75% subsample parameter was used alongside γ = 9 for aggressive pruning to mitigate overfitting. Raincloud plots were constructed using ‘ggplot2’ (version 3.4.4). To assess the efficacy of the biomarkers, receiver operating characteristic (ROC) curves were generated using the “pROC” package. Optimal cutoff points for the ROC curves were determined using Youden's index within the same package. Results for categorical variables were expressed as absolute and relative frequencies. In contrast, results for continuous variables were presented as means and standard deviations (SD) when the data were normally distributed, or as medians and interquartile ranges (IQR) when the data were not normally distributed. The Mann–Whitney U test was employed to analyze continuous variables. Correlation analysis was performed using Spearman's correlation analysis. A *p*-value < 0.05 was considered statistically significant.

## Results

### Baseline cohort characteristics

Our study design consisted of two major phases: a circulating microbial metagenomic sequencing phase, conducted on both serum and tissue samples, and a retrospective qRT-PCR-based serum training and validation phase (Figure S1). The characteristics of all study participants whose samples we used to develop and validate a cmDNA signature are summarized in Supplementary Tables 1–3.

### Distinct circulating microbial metagenomic profiles in patients with high-grade dysplasia or adenocarcinoma

**Figure 1.**
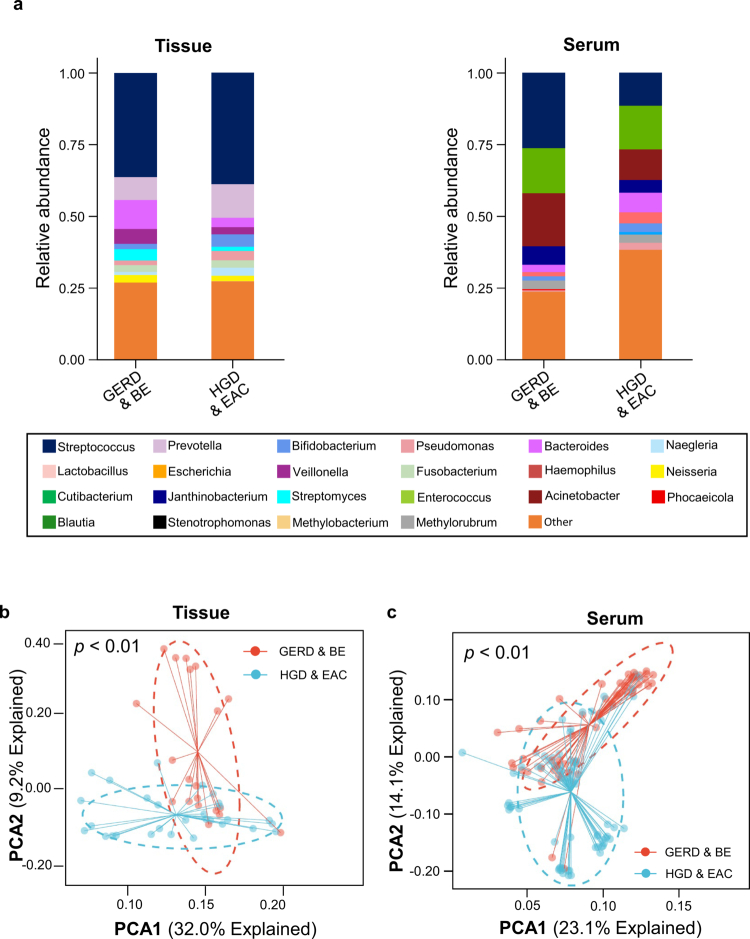
Relative abundance of top 10 microbiome taxa between the GERD/BE and the HGD/EAC groups in both tissue and serum samples. (a) Bar charts showing the component proportion of the top 10 most abundant genera between the GERD/BE and the HGD/EAC groups in both tissue and serum samples. The outer circle represents the HGD/EAC group, while the inner circle represents the GERD/BE group. (b) Principal component analysis (PCA) showed significant beta diversity between the GERD/BE and the HGD/EAC groups in both tissue and serum samples (*p* < 0.001). PCA1 and PCA2 values represent the top two principal coordinates, with blue representing the HGD/EAC group and red representing the GERD/BE group.

To identify a diagnostic cmDNA panel capable of distinguishing HGD/EAC from GERD/BE, we first performed metagenomic sequencing of tissue specimens from a cohort of 81 patients: 51 with EAC, 10 with HGD, 10 with BE, and 10 with GERD. We profiled the top ten bacterial genera in tissue specimens from both GERD/BE and HGD/EAC groups. Of these, six genera were shared between the two groups. These included *Streptococcus, Prevotella, Veillonella, Fusobacterium, Bacteroides, and Bifidobacterium*. However, the abundance of each genus differed between the two groups, with the remaining four genera in the top ten being unique to each group (Figure 1a, left). Additionally, we performed metagenomic sequencing analysis on paired serum specimens collected from the same 81 patients, along with an additional cohort of 13 cases with GERD, 27 with BE, 8 with HGD, and 5 with EAC (Figure S1). Similarly, in the serum species from both HGD/EAC and GERD/BE groups, seven of the top ten genera were shared between the two groups, with dominant genera including *Enterococcus, Pseudomonas, Bacteroides, Janthinobacterium, Streptococcus, Bifidobacterium, and Acinetobacter* (Figure 1b, right). Interestingly, three genera were shared between the tissue and serum phases, highlighting the tissue-specificity of cmDNA for the noninvasive detection of patients with HGD/EAC (Figure 1a). These findings highlight substantial differences in microbiome composition between the two disease groups, with overlapping microbial signatures shared between tissue and serum samples. PCA analyses of the relative abundance of genus DNA in each specimen revealed that the GERD/BE group could be distinguished from the HGD/EAC group in both tissue (Figure 1b) and serum (Figure 1c) specimens. Collectively, these data suggest that HGD/EAC exhibit unique diversity and microbial distance metrics compared to their corresponding control groups in both tissue and serum samples from patients with HGD and EAC, respectively.

### An increase in microbial diversity in the blood specimens of patients with EAC is a unique clinical feature

Compared with the control group (GERD/BE), microbial alpha-diversity was significantly lower in the HGD/EAC group in serum, at both the genus and species levels (Figure S2A, B). Evaluation of alpha-diversity included genus richness [represented by Chao1 (*p* < 0.01), good coverage (*p* = 0.234), Richness (*p* < 0.01), and ACE (*p* < 0.01)], as well as genera evenness [represented by Shannon (*p* < 0.01) and Simpson index (*p* = 0.234)]. Similarly, the evaluation of alpha-diversity encompassed richness [represented by Chao1 (*p* < 0.01), good coverage (*p* = 0.234), Richness (*p* < 0.01), and ACE (*p* < 0.01)], and evenness [represented by Shannon (*p* < 0.01) and Simpson index (*p* = 0.534)] in serum microbiome species. Interestingly, as esophageal disease progresses, from GERD, BE, and HGD to EAC, the genus- and species-level alpha diversity in serum samples gradually increases (Tables 1 and 2). These results confirm the occurrence of dysbiosis during the progression of malignant disease from GERD to BE to HGD and EAC.

**Table 1.** Multiple comparisons of *α*-diversity between GERD, BE, Dysplasia, and EAC in serum genera.

	GERD	BE	Dysplasia	EAC	*p*-value	Significance
ACE	50.59	74.10	78.52	89.29	0.00235	**
Chao1	39.59	64.32	71.64	80.36	0.0053	**
Goods coverage	0.77	0.81	0.87	0.83	0.543	ns
Richness	23.52	31.83	43.78	49.79	0.0164	*
Shannon	2.82	3.15	3.12	3.22	2.82E-05	****
Simpson	0.77	0.81	0.78	0.81	8.49E-06	****

Notes:

• *p*-values are based on Kruskal–Wallis tests.

• Significance: ns = not significant; *p <0.05 (*), <0.01 (**), <0.001 (***), <0.0001 (***).

**Table 2.** Multiple comparisons of *α*-diversity between GERD, BE, Dysplasia, and EAC in serum species.

	GERD	BE	Dysplasia	EAC	*p*-value	Significance
ACE	358.38	438.07	503.75	1124.76	8.8E-06	****
Chao1	318.95	406.1	400.23	1022.56	2.82E-05	****
Goods coverage	0.67	0.75	0.72	0.79	0.102	ns
Richness	137.83	215.97	147.83	450.51	0.00471	**
Shannon	4.63	4.77	4.95	5.34	2.82E-05	****
Simpson	0.89	0.85	0.84	0.92	1.34E-01	ns

Notes:

• *p*-values are based on Kruskal–Wallis tests.

• Significance: ns = not significant; *p <0.05 (*), <0.01 (**), <0.001 (***), <0.0001(****).

### Identification of potential cmDNA biomarkers for the early detection of HGD and EAC

**Figure 2.**
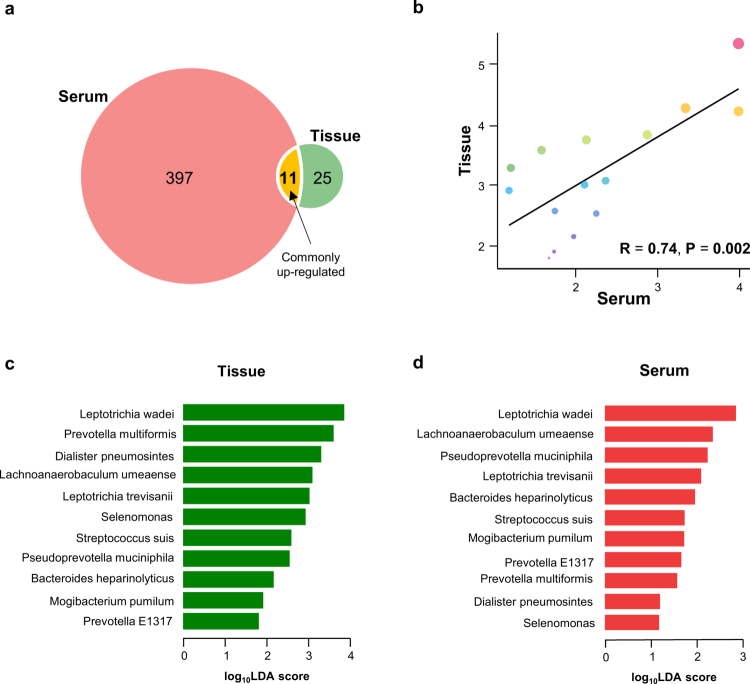
Taxonomic panels of GERD/BE and HGD/EAC microbiota. (a) Venn Diagram of upregulated microbial species in serum and tissue samples. A total of 397 species were uniquely upregulated in serum (red), while 25 species were upregulated in tissue (green). 11 species (yellow) showed consistent upregulation in both serum and tissue samples. (b) The co-relationship between the 15 candidates in tissues and serum phases. Scatter plot showing the correlation between upregulated microbial species in serum and tissue samples. A strong positive correlation was observed (R = 0.74, *p* = 0.002), suggesting that microbial species upregulated in serum are similarly elevated in tissue. (c, d) Linear discriminant analysis (LDA) reveals significant differences in abundances of bacterial taxa between the GERD/BE and HGD/EAC groups in both tissue and serum. Applying consistent filtering criteria (*p* < 0.05 and LDA > 1), we identified 11 potential microbiome candidates that distinguish the two groups. *p values are calculated by the Kruskal-Wallis test.*

In the discovery phase, we aimed to identify clinically relevant cmDNA as potential biomarkers for detecting patients with HGD/EAC. To this end, we used LEfSe to delineate differences in microbiome species between the controls and HGD/EAC patients. To pinpoint tissue-specific species for detecting HGD/EAC, we applied criteria of LDA > 1 and *p* < 0.05 across both tissue and serum phases. In the tissue phase, 36 candidates were upregulated in the HGD/EAC group. In comparison, 408 candidates exhibited an elevation in the serum phase (Figure 2a). We selected 11 candidates for further validation because they consistently showed upregulation in both the tissue and serum phases (Figure 2a). Correlation analysis of the 15 common species between tissue and serum revealed a strong positive correlation (r = 0.74, *p* < 0.05; Figure 2b). The relative abundance of *Leptotrichia wadei, Prevotella multiformis, Dialister pneumosintes, Lachnoanaerobaculum umeaense, Leptotrichia trevisanii, Selenomonas, Streptococcus suis, Pseudoprevotella muciniphila, Bacteroides heparinolyticus, Mogibacterium pumilum*, and *Prevotella* was significantly increased in the HGD/EAC groups (Figure 2c, d). These findings underscore a robust, tissue-derived microbial signature detectable in circulation, highlighting the promise of cmDNA as a noninvasive, clinically actionable biomarker panel for the early detection of HGD and EAC.

### Machine-learning powered development of a diagnostic cmDNA panel in the training cohort

**Figure 3.**
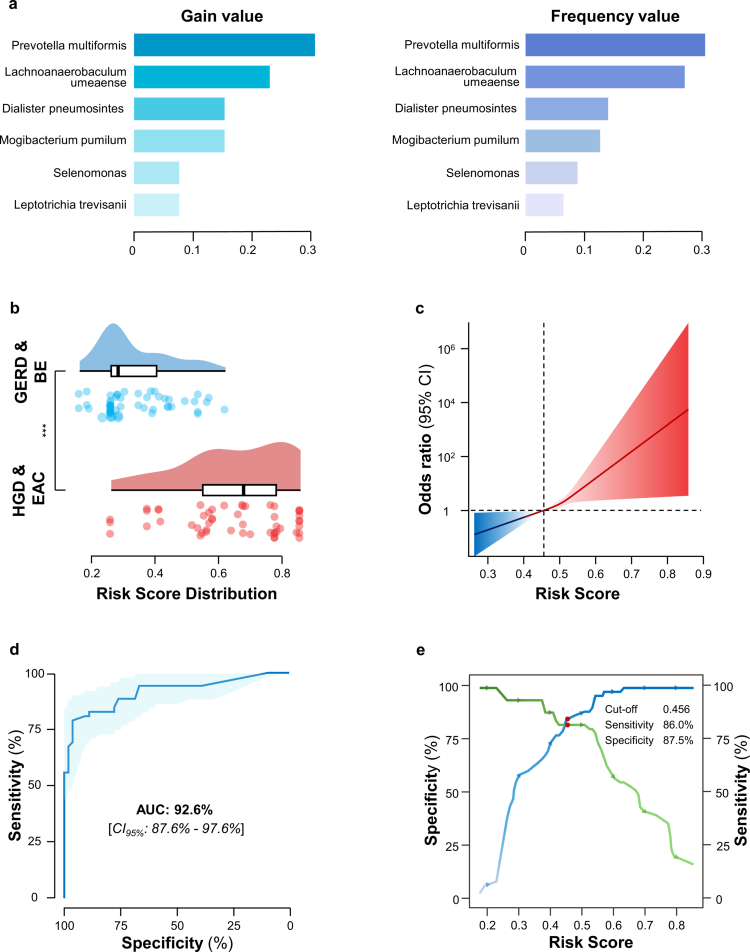
Development of a cmDNA biomarker panel in a clinical training cohort. (a) Bar chart showing the gain and frequency of each marker in the circulating microbial DNA biomarker panel. The green represents the gain for each candidate, which indicates the relative contribution of the specific feature to the XGBoost tree model, determined by assessing each feature's contribution across all trees. The blue represents the frequency of each candidate in the tree, indicating the percentage representing the relative number of times a particular feature occurs in the model's tree. (b) Raincloud plot showing risk score analyses based on risk prediction formulas for the GERD/BE and HGD/EAC groups in the training cohort. (c) The relationship between the cmDNA risk score and the odds ratio (95% CI) for diagnosing EAC or dysplasia. As the risk score increases, the odds ratio rises significantly, indicating a strong positive association. The shaded area represents the 95% confidence interval. (d) ROC curve analysis to examine the performance of 6 cmDNA-biomarker panels with AUC = 0.92. (e) The sensitivity (green) and specificity (blue) of the cmDNA panel across different risk scores for diagnosing EAC and dysplasia. The optimal cut-off point (risk score = 0.456) is highlighted, with corresponding sensitivity of 86.0% and specificity of 87.5%. ROC, receiver operating characteristics; AUC, area under the curve.

To further translate our sequencing-based discovery findings into a clinic-friendly blood-based assay, we sought to confirm whether the 11 prioritized candidates could be readily measured in blood using qRT-PCR. We first combined the AHN and JHU clinical samples, which had comparable numbers of patients with BE, HGD, and EAC, and then applied computer-based random sampling to divide them into training and validation sets. The division ensured that both sets included cases of BE, HGD, and EAC, with the training set approximately twice the size of the validation set. In the training cohort, four of the candidate markers had very low blood expression levels in more than 50% of specimens and were therefore excluded from subsequent analysis. The remaining seven candidates were abundantly expressed in blood and were adequate for analysis in the subsequent phases of our study. Based on these 7 markers, we developed a classifier for the training cohort using the XGBoost machine-learning algorithm and the expression levels of these candidates. We calculated SHapley Additive exPlanations (SHAP) and feature values to assess the performance of each candidate in the model. Our observations revealed that six of the seven biomarkers contributed significantly to the classifier (Figure S3A). We employed bar charts to visually represent the gain (Figure 3a, left) and frequency values (Figure 3a, right) of cmDNA contributing to our XGBoost classifier. Interestingly, biomarkers with higher gain and frequency values also appeared more frequently in the tree architecture and occupied higher positions (Figure S3B).

Next, we used the formula derived from the XGBoost classifier to calculate each patient's cmDNA risk score. As a result, the cmDNA panel yielded significantly higher risk scores in patients with HGD/EAC compared to those with BE (Mean risk score: BE, 0.39; HGD/EAC, 0.68; *p* < 0.001; Figure 3b). Importantly, this panel also demonstrated a linear association, with odds ratios (ORs) for HGD/EAC patients, showing a proportional increase in risk with higher risk score levels (*p* < 0.05; Figure 3c). The cmDNA-based liquid biopsy demonstrated high performance in distinguishing HGD/EAC from controls, with an AUC of 92.6% (CI 95%: 87.6%–97.6%), as shown in Figure 3d. Specifically, the plots of sensitivity and specificity versus probability cut-off points illustrated the performance of the cmDNA-based blood test in the training cohort (Figure 3e). At a cutoff of 0.456, determined by Youden's index, this blood-based test detected 86.0% of individuals with HGD and EAC, with a false-positive rate of 12.5% (Figure 3e). Taken together, these results indicate the highly promising potential of the cmDNA-based assay in identifying patients with HGD and EAC.

**Figure 4.**
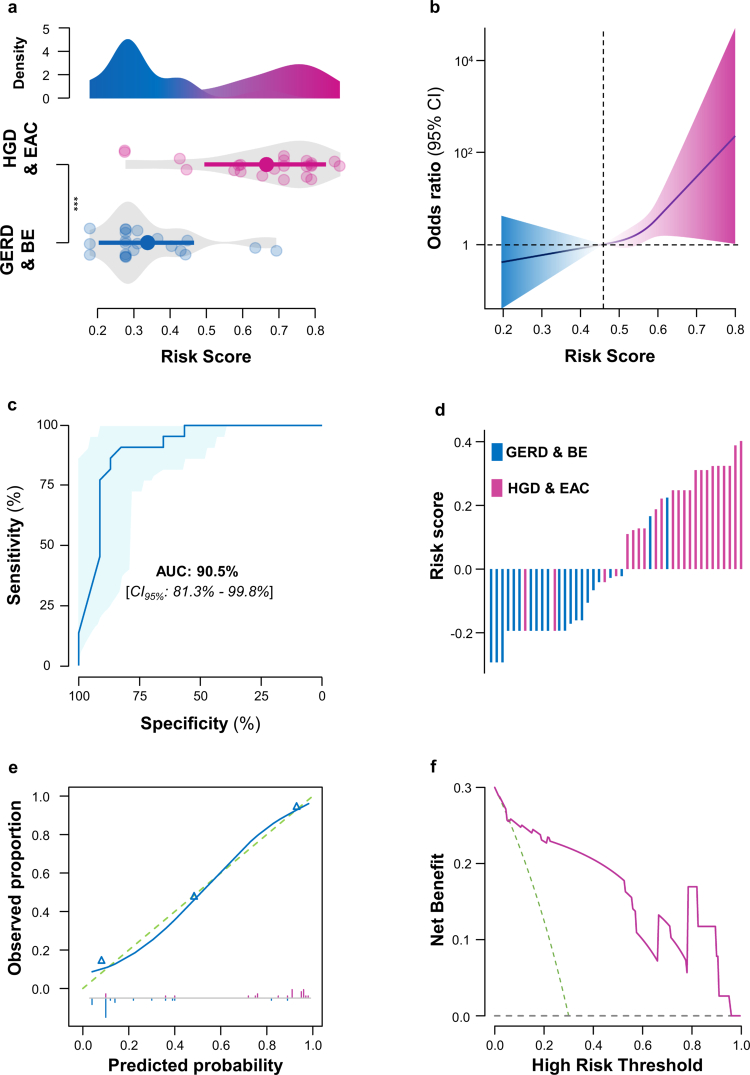
Performance evaluation of the 6-cmDNA panel in clinical validation cohort. (a) Density and violin plots showing differences in expression patterns between the GERD/BE and HGD/EAC groups in the validation cohort. The density distributions of the two groups exhibit clear distinctions with minimal overlap. (b) The relationship between the cmDNA risk score and the odds ratio (95% CI) for diagnosing EAC or dysplasia in the validation cohort. (c) ROC curve analysis was conducted to discern patients with HGD/EAC from those with GERD/BE, yielding an AUC of 0.91. ROC curves are accompanied by 95% confidence intervals. (d) Risk probability distribution plot in a training cohort of serum samples from patients with GERD/BE and HGD/EAC. (e) Calibration curve of 6-cm DNA panel in patients with HGD/EAC from the validation cohort. The dashed green line is the ideal line. The triangle sign indicates the grouped observations. The short line above and below the horizontal axis represents the positive and negative cases. (f) The decision curve shows the net benefit for the 6-cm DNA panel in patients with HGD/EAC from the validation cohort.

Successful testing of the cmDNA diagnostic assay in an independent clinical cohort. To further test the performance of the cmDNA-based panel for early detection of HGD/EAC, we evaluated the expression levels of six cmDNA biomarkers by qRT-PCR in a validation cohort comprising 22 BE patients and 23 HGD/EAC patients. We applied the XGBoost classifier to these patients using the same formula and cut-off values as in the training cohort. Density and violin plots showed a clear separation of cmDNA risk scores between the BE and HGD/EAC groups in the testing cohort, with significantly higher scores in the HGD/EAC group (Figure 4a). During the testing phase, the odds ratios for HGD/EAC disease also increased with higher cmDNA panel scores (*p* < 0.05; Figure 4b). Moreover, the cmDNA panel exhibited superior diagnostic performance, with an AUC value of 90.5% (CI 95%: 81.3%–99.8%) in detecting patients with HGD/EAC (Figure 4c), yielding robust results compared to the training cohort. In detail, the cmDNA panel correctly distinguished 20 out of 22 true BE patients and 19 out of 23 true HGD/EAC patients, with an accuracy of 82.6% (Figure 4d, Supplementary Table 5).

Additionally, calibration curve analysis confirmed that the panel's predicted probabilities closely aligned with the actual diagnoses for HGD/EAC patients (Figure 4e). Decision curve analysis (DCA) further demonstrated the clinical significance of the cmDNA panel for risk stratification in the testing cohort. As illustrated in the DCA curves, the combined biomarker panel provided a significantly higher net benefit than conventional strategies of treating all or none of patients, suggesting that this approach could reduce misdiagnosis and improve patient outcomes in clinical practice (Figure 4f). In summary, we successfully established and tested a cmDNA-based liquid biopsy assay for diagnosing patients with HGD/EAC from those with GERD/BE.

### The noninvasive cmDNA diagnostic panel demonstrates robust clinical significance in identifying patients with dysplasia

Subsequently, we performed subgroup analyses to further assess the performance of our cmDNA panel for diagnosing HGD and EAC in the testing cohort. The cmDNA panel levels were significantly higher in patients with EAC or HGD than in individuals with BE in the testing cohort (*p* < 0.01; Figure S4A). The diagnostic potential of the cmDNA panel was systematically assessed, yielding AUC values of 88.3% for HGD (Figure S4B) and 91.3% for EAC (Figure S4C). Specifically, the panel achieved 90.9% specificity for both HGD and EAC, and corresponding sensitivities of 83.3% for HGD and 81.3% for EAC. The overall accuracy was 89.3% for HGD and 86.8% for EAC, respectively (Figure S4D, Supplementary Table 6). These results demonstrated the effectiveness of our cmDNA assay in diagnosing patients with both HGD and EAC, positioning it as a promising biomarker for identifying individuals at high risk of HGD and those with EAC. These findings underscore the potential of this cmDNA panel for early detection and intervention at the pre-malignant stage.

## Discussion

The incidence of EAC is rising by 4% to 10% annually in many Western countries, making it one of the fastest-growing cancers in developed nations over the past 30 years.^21^,^22^ Although advancements in treatment have modestly improved survival rates, half of the patients still succumb within a year of diagnosis.^23^ The primary challenge lies in the inadequacy of current EAC screening measures, which are insufficient for detecting precancerous and early-stage tumors. BE is the only known precursor lesion of EAC.^24^ Early screening and continuous monitoring of BE are essential for detecting HGD and early-stage EAC.^25^ When dysplasia or early-stage EAC is identified during surveillance, endoscopic resection or ablation therapy can be used to prevent EAC progression.^26^

Emerging technologies with the potential to enhance the early detection of EAC and its precancerous lesions include capsule endoscopy, analysis of volatile organic compounds in exhaled breath, and minimally invasive capsule-delivered sponges.^27^ Notably, cytosponge-TFF3 testing could offer a feasible, safe, and acceptable screening method for the early diagnosis of treatable dysplasia and early cancer through a patient-swallowed sponge that collects esophageal epithelial cells for analysis.^28^ However, blood-based biomarkers for diagnosing HGD and early-stage EAC, such as circulating cell-free DNA (cfDNA) for liquid biopsies, have shown limited success, with reported sensitivities of less than 20% in tests like Galleri and CancerSEEK.^29^,^30^

Growing evidence suggests alterations in the diversity and function of the oral and esophageal microbiomes in patients with BE and EAC.^31–33^ Non-invasive microbiome panels are emerging as potential diagnostic tools for BE and EAC. In a study with 49 participants (17 controls and 32 BE patients), saliva samples analyzed via 16S rRNA sequencing effectively distinguished BE patients from controls, with an AUC of 0.94.^34^ Importantly, during the sequential progression from GERD to BE, then to HGD and ultimately EAC, studies have shown that detectable microbial compositional shifts are already present at the BE stage, suggesting that specific microbiome alterations may precede or accompany histological changes. This highlights the potential importance of microbiome-based biomarkers for anticipating malignant transformation at an earlier stage. Recently, cmDNA has emerged as a promising source of microbiome DNA, drawing considerable research interest.^16^,^35^,^36^ Chen et al. developed a machine learning model using cmDNA for the early detection of lung cancer (LC), which achieved a high sensitivity of 86.5% in distinguishing early-stage LC from non-cancerous subjects.^16^ Our research group has also developed a cmDNA panel for the early diagnosis and prognosis of EAC.^37^ However, previous studies have not clarified the origin of cmDNA. In this study, we aimed to explore the properties and potential of cmDNA by analyzing both tissue specimens and paired serum samples. We categorized samples from patients with GERD, BE, HGD, and EAC into two groups: one consisting of GERD and BE patients under continuous surveillance, and the other comprising HGD and EAC patients at high risk of developing advanced disease. Through this analysis, we identified differences in cmDNA characteristics between these groups. Subsequently, we trained and validated a cmDNA diagnostic panel to detect early-stage EAC and identify individuals at high risk of EAC progression.

In this study, we found distinct cmDNA profiles in GERD/BE and HGD/EAC patients. The HGD/EAC groups exhibited greater microbiota diversity. Alpha diversity, which is influenced by dietary patterns, health conditions, and treatments such as antibiotics and PPIs, increased with the progression of esophageal disease (Tables 1 and 2). Through biomarker discovery and expression profiling, we identified 11 cmDNA markers that were significantly upregulated in patients with HGD/EAC compared to those with GERD/BE, and these markers were detected in both tissue and serum samples. Notably, many of these 11 cmDNA candidates are implicated in cancer development. For instance, *Selenomonas*, commonly associated with *Fusobacterium* and extensively studied in cancer research, has been detected in distal metastases of colorectal cancer.^38^ *Dialister pneumosintes* is one of the bacterial taxa enriched in GC,^39^ and *Leptotrichia trevisanii* has been used in a microbial panel to distinguish oral squamous cell carcinoma (OSCC) recurrences from primary OSCC with high accuracy (accuracy = 0.96).^40^ *Streptococcus suis* has also been reported as a potential noninvasive screening marker for GC.^41^ We then developed a cmDNA panel for diagnosing patients with EAC and dysplasia using the XGBoost machine learning model. This model accurately distinguished HGD/EAC patients from GERD/BE subjects, achieving a mean AUC of 92.6%, with 87.5% specificity and 86.0% sensitivity. Using the same algorithm and cutoff values, we validated the panel in a separate cohort, achieving robust performance with an AUC of 90.5%, sensitivity of 82.6%, and specificity of 90.9%. Furthermore, our panel accurately identified patients with either EAC (AUC = 91.3%) or HGD (AUC = 88.3%). These findings highlight the importance of microbial dysbiosis in EAC progression and suggest that integrating multi-omics data—such as combining microbiome profiles with metabolomic,^42^ transcriptomic,^43^,^44^ or epigenomic signatures^45^ —could further elucidate the mechanistic links between microbial alterations and tumorigenesis. Multi-omics approaches may also help identify additional predictive biomarkers,^46^ refine risk stratification, and provide a more comprehensive understanding of how microbial communities interact with host pathways during disease progression.

Despite the promising results, this study has some limitations that require attention. Although our cmDNA-based model demonstrated high accuracy in detecting EAC and identifying individuals at high risk, the sample size of patients with dysplasia was limited. Furthermore, our validation cohort included only HGD cases, which is insufficient to fully characterize the cmDNA signature associated with EAC progression. Therefore, further validation using a larger cohort with a broader spectrum of dysplastic lesions is necessary to confirm the model's utility for early detection.

## Conclusions

Our study provides valuable insights into the significant alterations in cmDNA profiles, which could serve as a promising resource for developing a liquid biopsy for the early detection of patients with esophageal neoplasia (HGD and EAC). In clinical practice, such an assay could function as a triage tool to efficiently identify high-risk individuals who require further endoscopic evaluation, potentially improving patient outcomes. We acknowledge the limitations of our sample size and the potential influence of confounding factors. To advance clinical translation, future studies should focus on larger, multicenter, prospective trials to validate these findings and explore integrating cmDNA testing into routine screening and monitoring strategies.

## Supplementary Material

Supplementary material Supplementary data

## Data Availability

The datasets used and/or analyzed during the current study are available with the following identifier (DOI: 10.6084/m9.figshare.29270888), as well as at the following website (https://figshare.com/s/7c3991c3883264338706).

## Abbreviations

BE: Barrett's esophagus
cfDNA: cell-free DNA
cmDNA: circulating microbial DNA
DCA: decision curve analysis
EAC: esophageal adenocarcinoma
EC: esophageal cancer
FC: fold change
GERD: gastroesophageal reflux disease
HGD: high-grade dysplasia
LGD: low-grade dysplasia
LEfSe: linear discriminant analysis effect size
PCA: principal component analysis
qRT-PCR: quantitative reverse transcription polymerase chain reaction
LC: lung cancer
OR: odds ratio
