# Supplementary material for: A machine-learning informed circulating microbial DNA signature for early diagnosis of esophageal adenocarcinoma
Source: Gut Microbes. 2025 Dec 24;18(1):2604334. doi: 10.1080/19490976.2025.2604334 (PMC12758224; doi:10.1080/19490976.2025.2604334)
Supplement: Supplementary material — Supplementary data [file KGMI_A_2604334_SM2024.docx]

**Supplementary Materials:**

**Supplement Figure S1:** Study design for identification of a circulating microbial DNA biomarker panel for early detection of EAC.


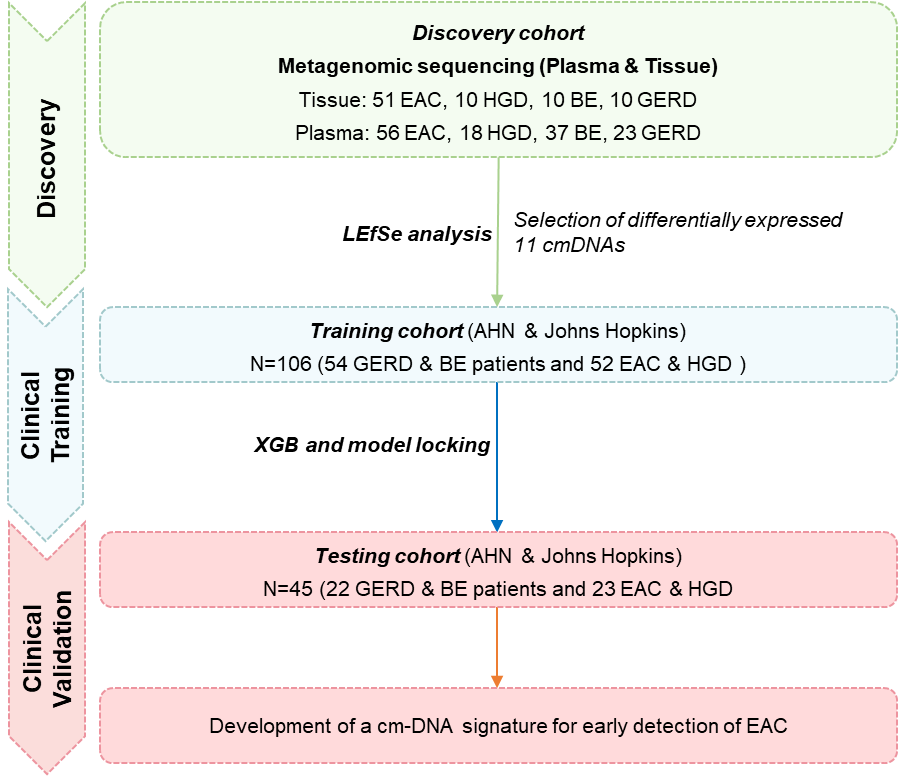


**Supplement Figure S2:** (**A**) The comparison of microbiota alpha diversity at the genus level between GERD/BE and HGD/EAC, including species richness [as represented by Chao1 (p < 0.01), good coverage (*p* = 0.234), Richness (*p* < 0.01), and ACE (*p* < 0.01)], and evenness [as represented by Shannon (*p* < 0.01) and Simpson index (*p* = 0.234)]. (**B**) The comparison of microbiota alpha diversity at the species level between GERD/BE and HGD/EAC, including species richness (as represented by Chao1 (*p* < 0.01), good coverage (*p* < 0.01), Richness (*p* < 0.01), and ACE (*p* < 0.01)), and evenness (as represented by Shannon (*p* < 0.01) and Simpson index (*p* = 0.534)) revealed that the HGD/EAC group exhibited higher diversity, richness, and evenness compared to the control group at both genus and species level.

**
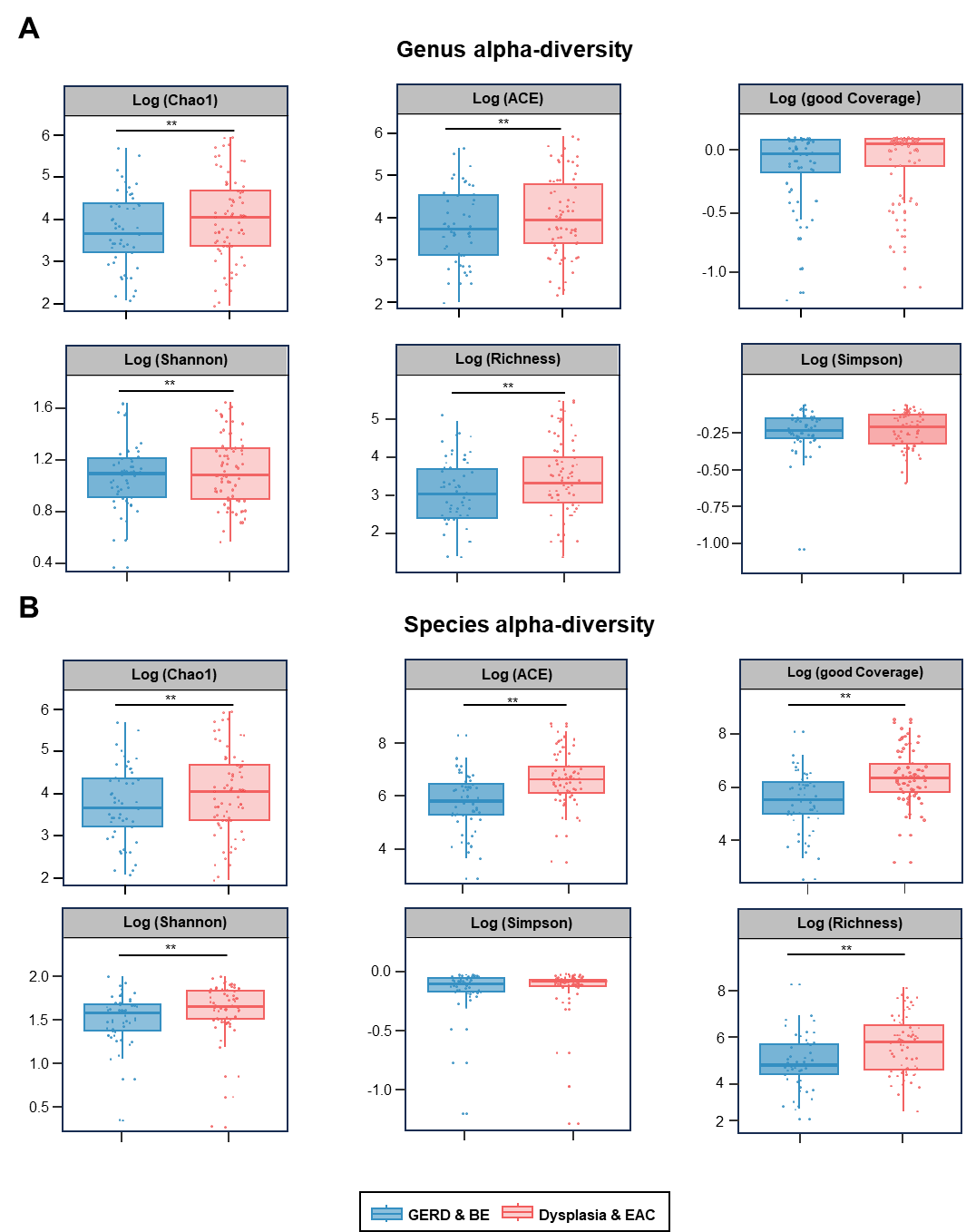
**


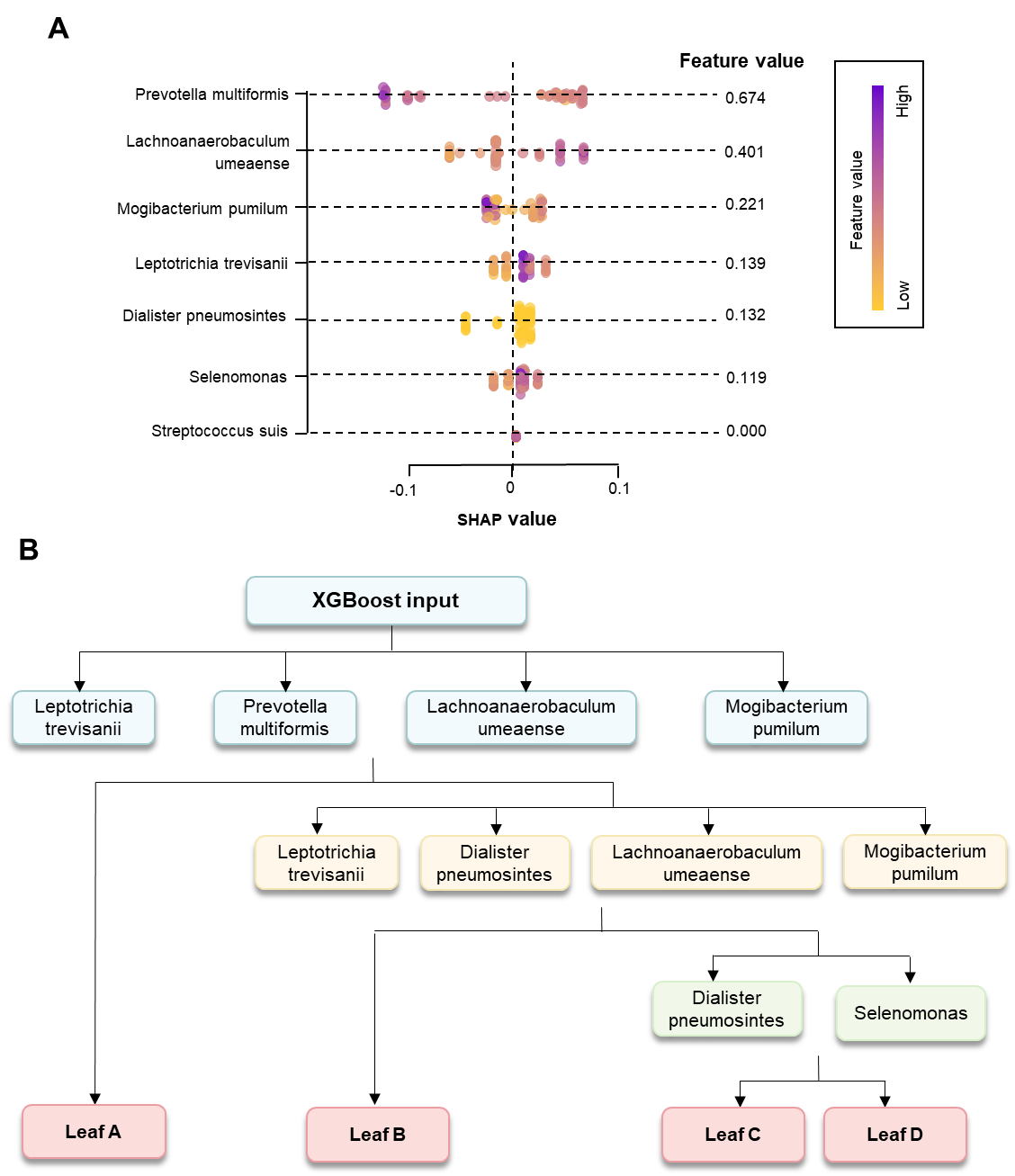
**Supplement Figure S3:** (**A**) The SHAP summary plot depicts the relationship between the values of 7 cmDNA biomarker features and their SHAP values. Purple and yellow colors indicate high and low feature values, respectively. (**B**) A schematic of an XGBoost machine learning gradient-boosted trees algorithm. The XGBoost model was trained over 5000 rounds of boosting using gbtrees, with a maximum tree depth restricted to three splits. Additionally, a 75% subsample parameter was utilized, alongside γ=9 for aggressive pruning to mitigate overfitting risks. The output layer displays the event predicted by the cmDNA panel based on the training data.

**Supplement Figure S4:** Performance evaluation of the 6-cmDNA panel to identify patients with either EAC or dysplasia. (**A**) The cmDNA-based risk score was evaluated in patients from the validation cohort, including those with GERD/BE, dysplasia, and EAC. (**B**) ROC curve analysis demonstrates the 6-cmDNA panel performance between patients with dysplasia (n = 6) and GERD/BE controls (n = 22) in the validation cohort. (**C**) ROC curve analysis demonstrates the 6-cmDNA panel. (**D**) The sensitivity, specificity and accuracy of 6-cmDNA panel in detection of patients with dysplasia (n = 6) and GERD/BE controls (n = 22) in the validation cohort.

**
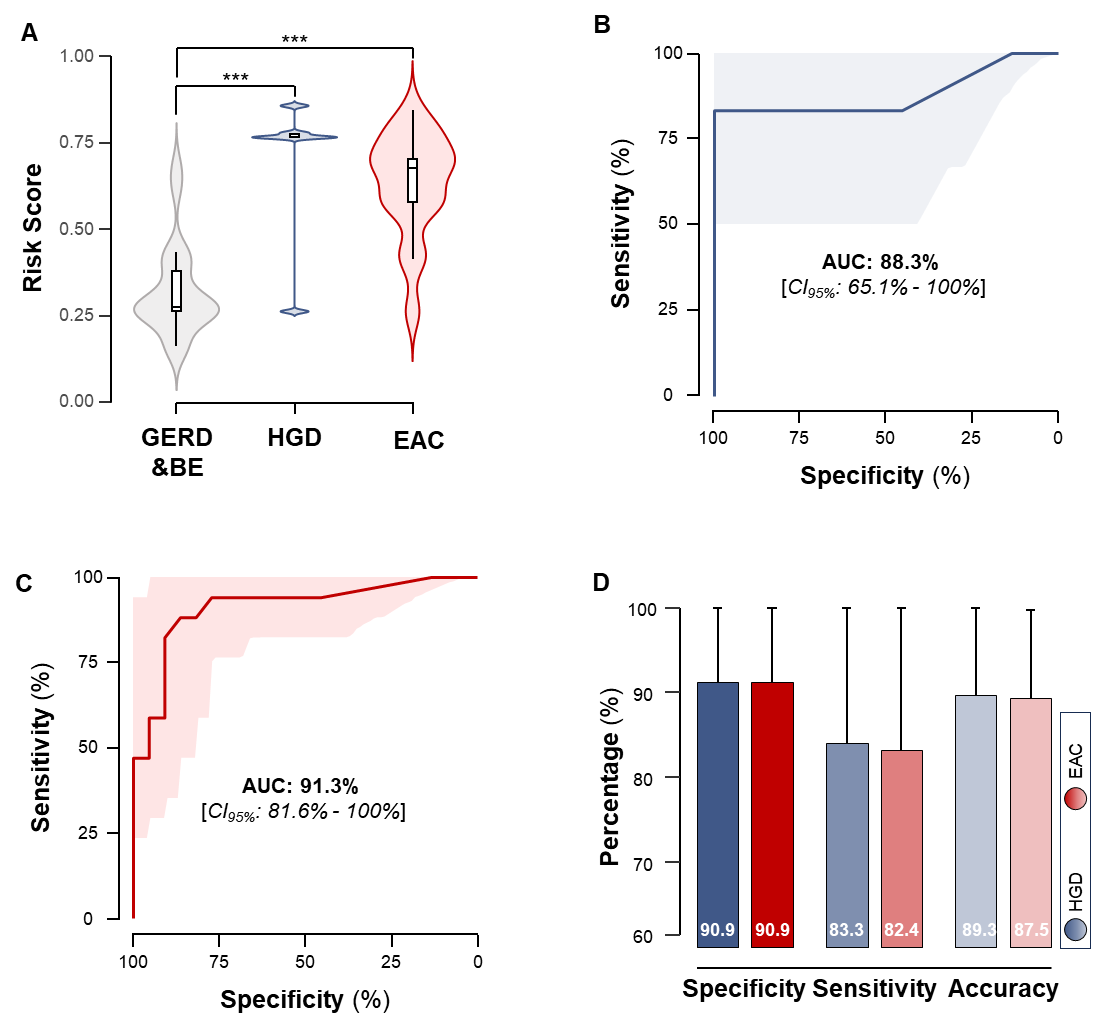
**

**Supplementary Table 1: Clinical information for discovery phase (Tissue phase)**

| **Characteristics** | GERD | BE | Dysplasia | EAC |
| --- | --- | --- | --- | --- |
|  | (n = 10) | (n = 10) | (n = 10) | (n = 51) |
| **Age (years)** | | | | |
| Median (range) | 65.5 (35-76) | 68 (25-95) | 74 (63-81) | 67 (44-95) |
| **Gender** | | | | |
| Male | 2 | 7 | 10 | 43 |
| Female | 9 | 3 | 0 | 9 |
| **Clinical TNM Stage** | | | | |
| 1-2 |  |  |  | 18 |
| 3- 4 |  |  |  | 33 |
| **Nodes** | | | | |
| Positive |  |  |  | 25 |
| Negative |  |  |  | 20 |
| Metastasis |  |  |  | 6 |
| **Recurrence** | | | | |
| No |  |  |  | 33 |
| Yes |  |  |  | 17 |
| Unknown |  |  |  | 1 |
| **Progression to metastasis** | | | | |
| No |  |  |  | 19 |
| Yes |  |  |  | 28 |
| Unknown |  |  |  | 4 |
| **PPI Use** | | | | |
| No | 8 | 7 | 7 | 24 |
| Yes | 2 | 3 | 3 | 27 |
| **Antibiotic Use** | | | | |
| No | 10 | 7 | 10 | 42 |
| Yes |  | 3 |  | 9 |
| **Treatment** | | | | |
| **EAC** | | | | |
| Carboplatin and Paclitaxel |  |  |  | 23 |
| FOLFOX (with or without radiation) |  |  |  | 6 |
| Other chemotherapy regiments |  |  |  | 7 |
| Nivolumab |  |  |  | 2 |
| Radiotherapy |  |  |  | 2 |
| Esophagectomy |  |  |  | 30 |
| Gastrectomy |  |  |  | 2 |
| EMR |  |  |  | 5 |
| **GERD** | | | | |
| Proton Pump Inhibitor (PPI) | 4 |  |  |  |
| Nissen fundoplication | 6 |  |  |  |
| Palliative |  |  |  | 11 |
| **BE** | | | | |
| Proton Pump Inhibitor (PPI) |  | 2 |  |  |
| Nissen fundoplication |  | 3 |  |  |
| LINX® |  | 2 |  |  |
| Radiofrequency Ablation (RFA) |  | 2 |  |  |
| **Dysplasia** | | | | |
| Radiofrequency Ablation (RFA) |  |  | 5 |  |
| Endoscopic Mucosal Resection (EMR) |  |  | 3 |  |
| RFA + EMR |  |  | 1 |  |
| Esophagectomy |  |  | 1 |  |
| **Survival** | | | | |
| Dead | 0 | 1 | 0 | 38 |
| Alive | 10 | 9 | 10 | 13 |
| Overall survival (OS) | | | | |
| < 24 months |  |  |  | 22 |
| ≥ 24 months |  |  |  | 37 |

Abbreviation: GERD, Gastroesophageal reflux disease; BE, Barret esophagus; EAC, Esophageal carcinoma; TNM: Tumor, Nodule, Metastasis.

**Supplementary Table 2: Clinical information for discovery phase (Serum phase)**

| Characteristics | GERD | BE | Dysplasia | EAC |
| --- | --- | --- | --- | --- |
|  | (n = 23) | (n = 37) | (n = 18) | (n = 56) |
| Age (years) | | | | |
| Median (range) | 63 (28-90) | 60 (23-95) | 72.5 (38-85) | 67 (44-95) |
| Gender | | | | |
| Male | 5 | 20 | 15 | 46 |
| Female | 18 | 17 | 3 | 10 |
| Clinical TNM Stage | | | | |
| 1-2 |  |  |  | 19 |
| 3- 4 |  |  |  | 37 |
| Nodes | | | | |
| Positive |  |  |  | 28 |
| Negative |  |  |  | 22 |
| Metastasis |  |  |  | 9 |
| Recurrence | | | | |
| No |  |  |  | 33 |
| Yes |  |  |  | 17 |
| Unknown |  |  |  | 1 |
| Progression to metastasis | | | | |
| No |  |  |  | 20 |
| Yes |  |  |  | 31 |
| Unknown |  |  |  | 1 |
| PPI Use | | | | |
| No | 17 | 33 | 15 | 29 |
| Yes | 4 | 4 | 3 | 27 |
| Unknown | 2 |  |  |  |
| Antibiotic Use | | | | |
| No | 23 | 34 | 18 | 47 |
| Yes | 0 | 3 | 0 | 9 |
| Treatment | | | | |
| EAC | | | | |
| Carboplatin and Paclitaxel |  |  |  | 26 |
| FOLFOX (with or without radiation) |  |  |  | 1 |
| Other chemotherapy regiments |  |  |  | 7 |
| Nivolumab |  |  |  | 2 |
| Radiotherapy |  |  |  | 2 |
| Esophagectomy |  |  |  | 30 |
| Gastrectomy |  |  |  | 2 |
| EMR |  |  |  | 5 |
| GERD | | | | |
| Proton Pump Inhibitor (PPI) | 4 |  |  |  |
| Nissen fundoplication | 10 |  |  |  |
| Palliative | 0 |  |  | 11 |
| LINX® | 2 |  |  |  |
| BE | | | | |
| Proton Pump Inhibitor (PPI) |  | 4 |  |  |
| Nissen fundoplication |  | 12 |  |  |
| LINX® |  | 6 |  |  |
| Radiofrequency Ablation (RFA) |  | 2 |  |  |
| Dysplasia | | | | |
| Radiofrequency Ablation (RFA) |  |  | 6 |  |
| Endoscopic Mucosal Resection (EMR) |  |  | 3 |  |
| RFA + EMR |  |  | 3 |  |
| Esophagectomy |  |  | 1 |  |
| Survival | | | | |
| Dead | 0 | 1 | 1 | 42 |
| Alive | 23 | 9 | 10 | 14 |
| Overall survival (OS) | | | | |
| < 24 months |  |  |  | 26 |
| ≥ 24 months |  |  |  | 37 |

Abbreviation: GERD, Gastroesophageal reflux disease; BE, Barret esophagus; EAC, Esophageal carcinoma; TNM: Tumor, Nodule, Metastasis.

**Supplementary Table 3: Clinical information for training and validation phases**

| **Clinicopathological characteristics enrolled patients in training and validation cohort** | | |
| --- | --- | --- |
|  | Training cohort (n = 106) | Validation cohort (n = 45) |
| **Disease group (52/106; 23/45)** | | |
| Patients with HGD, n (%) | 7(17.1%) | 5 (0%) |
| Patients with EAC, n (%) | 45 (31.6%) | 18(50.7%) |
| Age, median, SD | 67 (± 9.1) | 65 (± 15.6) |
| Gender, n (%) | | |
| Male | 39/52 (75.0%) | 17 (73.9 %) |
| Female | 13/52 (25.0%) | 6 (26.1%) |
| Tumor stage in EAC group, n (%) | | |
| I | 5/45 (11.1%) | 2/18 (11.1%) |
| II | 3/45 (6.7%) | 0/18 (0.0%) |
| III | 18/45 (40.0%) | 5/18 (27.8%) |
| IV | 4/45 (8.9%) | 3/18 (16.7%) |
| NA | 15/45 (33.3%) | 8/18 (44.4%) |
| Study site, n (%) | | |
| AHN cohort | 26 (50%) | 11 (47.8%) |
| JHU cohort | 26 (50%) | 12 (52.2%) |
| **Control group (54/106; 22/45)** | | |
| Patients with GERD | 20 (0%) | 3 (0%) |
| Patients with BE, n (%) | 34 (51.3%) | 19 (49.3%) |
| Age, median (SD) | 67 (±14.8) | 62 (±14.2) |
| Gender, n (%) | | |
| Male | 34 (56.4%) | 14 (63.6%) |
| Female | 20 (43.6%) | 8 (36.4 %) |
| Study site, n (%) | | |
| AHN cohort | 26 (48.1%) | 13 (59.1%) |
| JHU cohort | 28 (51.9%) | 9 (40.9) |

Abbreviation: GERD, Gastroesophageal reflux disease; BE, Barret esophagus; EAC, Esophageal carcinoma.

**Supplementary Table 4: List of the Primers Used in This Study**

| **Primer** | **Sequence** |
| --- | --- |
| Streptococcus suis forward | ATCATGCCCCTTATGACCTG |
| Streptococcus suis reverse | TCCGAACTGAGACTGGCTTT |
| Dialister pneumosintes forward | GACGATCAGTAGCCGGTCTG |
| Dialister pneumosintes reverse | ACCCTTTCGTCCCGAATCAC |
| Selenomonas sputigena forward | ACGGTCGCAAGACTGAAACT |
| Selenomonas sputigena reverse | ACTCAATGTCAAGCCCTGGT |
| Bacteroides heparinolyticus forward | CGCAACCCTTATCCACAGTT |
| Bacteroides heparinolyticus reverse | GTGCTGATTTGACGTCATCC |
| Leptotrichia wadei forward | GTAACGCGTAAGGGACTTGC |
| Leptotrichia wadei reverse | TCTCCTGCAGCATTCCTTTT |
| Leptotrichia trevisanii forward | GCAAGGCTGAAACTCAAAGG |
| Leptotrichia trevisanii reverse | GCATACGCAGGATGTCAAGA |
| Lachnoanaerobaculum umeaense forward | AAGCAACGCGAAGAACCTTA |
| Lachnoanaerobaculum umeaense reverse | ACAACCATGCACCACCTGTA |
| Mogibacterium pumilum forward | AACTTCTACGGAAGGCAGCA |
| Mogibacterium pumilum Reverse | TACAACCCGAAGCCTTCATC |
| Prevotella multiformis forward | GCATGAATAAGGACCGGCTA |
| Prevotella multiformis Reverse | GCCTACGCTCCCTTTAAACC |
| Pseudoprevotella muciniphila forward | TTGGGTTTAAAGGGAGCGTA |
| Pseudoprevotella muciniphila Reverse | TGTGCGCACTCAAGATAACC |
| 16S-341 forward | CCTACGGGAGGCAGCAG |
| 16S-534 Reverse | ATTACCGCGGCTGCTGG |
|  |  |

**Supplementary Table 5. The diagnostic performance of cmDNA signature at varying sensitivity thresholds for detection EAC and HGD.**

|  | AUC (95%CI) | Sensitivity (95%CI)  @cutoff (0.456) ^a^ | Specificity (95%CI)  cutoff (0.456) ^a^ |
| --- | --- | --- | --- |
| EAC, % | 91.3 (81.6-100.0) | 81.3 (62.5-100.0) | 90.9 (77.3-100) |
| HGD, % | 88.3 (65.1-100.0) | 83.3 (50.0-100.0) | 90.9 (77.3-100) |

Sens: Sensitivity; Spec: Specificity; a: The cutoff value was determined from the training cohort.

**Supplementary Table 6. The diagnostic performance of cmDNA signature at varying sensitivity thresholds for detection in training and validation cohort.**

|  | AUC (95%CI) | Sensitivity (95%CI)  @cutoff (0.456) ^a^ | Specificity (95%CI)  cutoff (0.456) ^a^ |
| --- | --- | --- | --- |
| Training cohort, % | 92.6 (87.6-97.6) | 82.7 (73.1-92.3) | 87.4 (77.8-94.4) |
| Validation cohort, % | 90.5 (81.3-99.8) | 68.0 (48.0-88.0) | 85.0 (70.0-100.0) |

Sens: Sensitivity; Spec: Specificity; a: The cutoff value was determined from the training cohort.
